# Cutaneous Side Effects of Targeted Therapy and Immunotherapy for Advanced Melanoma

**DOI:** 10.1155/2018/5036213

**Published:** 2018-12-30

**Authors:** Irene Russo, Ludovica Zorzetto, Vanna Chiarion Sileni, Mauro Alaibac

**Affiliations:** ^1^Unit of Dermatology, University of Padua, Via Gallucci 4, 35128 Padova, Italy; ^2^Istituto Oncologico Veneto I.R.C.C.S., Via Gattamelata 64, 35128 Padova, Italy

## Abstract

Melanoma is one of the most fatal cancers, and its incidence is increasing worldwide. Thanks to the better understanding of the molecular mechanisms involved in the pathogenesis of melanoma, recently new targeted agents have been developed. In this article, we review the current state of knowledge of clinical presentation, mechanisms, and management of the most common cutaneous side effects observed during treatment with targeted and immunological therapies approved for advanced melanoma. We include discussion of BRAF/MEK inhibitors and immune-checkpoint inhibitors, notably CTLA-4 and PD-1 inhibitors.

## 1. Introduction

Melanoma is one of the most fatal cancers, and its incidence is increasing worldwide. Metastatic melanoma has a poor prognosis representing about 90% of skin cancer mortality. In the recent past, the therapy of metastatic melanoma was based only on dacarbazine, an alkylating chemotherapy agent, which has not shown improvement of overall survival [1]. Classical chemotherapy has cytotoxic and antiproliferative effects which give rise to well-known cutaneous adverse events (AEs). More recently, thanks to the better understanding of the molecular mechanisms involved in the pathogenesis of melanoma, new agents have been developed. In particular, inhibitors of the cytoplasmic serine/threonine kinase BRAF and the immune-checkpoint targeted agents, anti-cytotoxic T-lymphocyte-associated antigen 4 (CTLA-4), and anti-programmed cell death 1 (PD-1) inhibitors have been approved for the treatment of advanced melanoma. With the introduction of these novel therapies, a new spectrum of skin side effects has emerged. Management of these cutaneous AEs is very important for treatment adherence and patient quality of life. In this article, we review the current state of knowledge of clinical presentation, mechanisms, and management of the most common cutaneous side effects observed during treatment with targeted and immunological therapies approved for advanced melanoma. The main skin adverse events and their management are summarized in Table 1.

## 2. Targeting the BRAF/MEK Pathway

**Figure 1:**
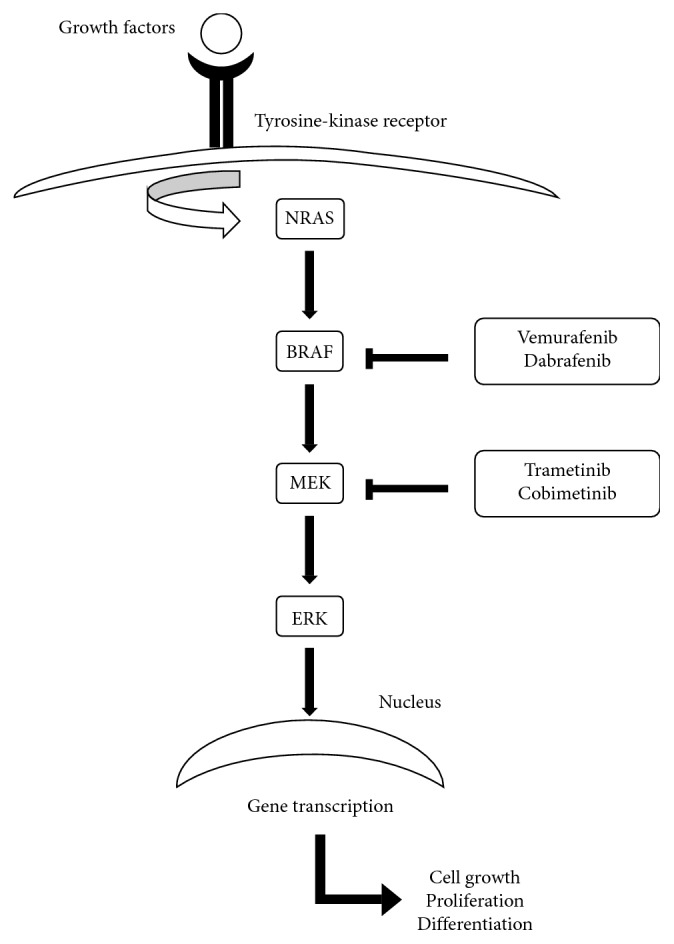
MAPK signalling pathway and its inhibitors.

Genetic alterations resulting in a constitutive activation of mitogen-activated protein kinase (MAPK) pathway occur in almost all cases of melanoma. The most frequent mutation is the BRAF mutation which has been found in about 50% of melanomas [2–4]. BRAF is a component of the RAS-RAF-MEK-ERK signalling pathway. The constitutive activation of mutated BRAF leads to an uncontrolled signal transduction of the MAPK pathway causing an increased proliferation, reduced apoptosis, and enhanced invasiveness [2, 3]. Since the discovery of BRAF mutations in cutaneous melanoma, nonselective and selective pharmacological agents have been developed to inhibit this target [5] (Figure 1).

The first selective BRAF inhibitor approved by FDA and EMA in 2011 for the treatment of patients with metastatic or unresectable melanoma was vemurafenib [2, 6], followed by dabrafenib which was approved by FDA and EMA in 2013 [7]. The adding inhibition of MEK, downstream of BRAF, has been reported to improve the duration of the therapeutic effect of BRAF inhibitors [8]. Two BRAF-MEK inhibitor combinations (vemurafenib-cobimetinib and dabrafenib-trametinib) are currently regarded as treatment options for metastatic or unresectable melanoma [9, 10], and in the near future, a new combination therapy (encorafenib-binimetinib) is expected to emerge as a valuable alternative to established BRAF-MEK combinations [11].

### 2.1. Cutaneous Side Effects of BRAF-MEK Inhibitors

Cutaneous side effects of BRAF inhibitors are very common and usually occur within days of the undergoing treatment. Skin toxicity induced by BRAF inhibitors is similar to that observed with EGFR inhibitors as BRAF represents a downstream mediator of EGFR signalling which is a critical regulator of epidermal homeostasis [12]. Although skin toxicity induced by BRAF inhibitors may significantly reduce the quality of life of these patients, it is generally managed with dose adjustment and supportive treatments. Furthermore, the combination of a MEK inhibitor with a BRAF inhibitor has been reported to induce less skin toxicity compared to the BRAF inhibitor monotherapy approach [9, 12–17].

Biological results demonstrate that BRAF inhibitors promote paradoxical activation of the MAPK pathway in cells that do not carry BRAF mutation leading to keratinocyte proliferation and hyperproliferative skin lesions which are the most common side effect of BRAF inhibitors [18]. A concurrent inhibition downstream of RAF with a MEK inhibitor preventing the pathway activation results in the significant reduction in cutaneous side effects [19].

The profile of skin toxicity associated with MEK inhibitors differs from that occurring during BRAF inhibitors therapy. Squamoproliferative skin lesions developed in patients treated with BRAF inhibitors have not been reported during treatment with MEK inhibitors. The typical rash (papulopustular or acneiform) observed during MEK inhibitors agents is different from the rash (hyperkeratotic maculopapular) caused by BRAF inhibitors [20].

### 2.2. Rash

Skin rash has been reported in patients treated both with BRAF and MEK inhibitors. Skin rash is mostly seen in patients under treatment with BRAF inhibitors (64–71% with vemurafenib and up to 18% with dabrafenib) [21]. It usually occurs 2 weeks after initiation of therapy and develops on the face/neck, trunk, and extremities. Many subtypes of rash have been described: macular, maculopapular, papular, and papulopustular. The maculopapular rash, also known as morbilliform rash, is the most common during BRAF inhibitors therapy. It is characterized by macules (flat) and papules (elevated) frequently involving the upper trunk, expanding centripetally and associated with pruritus. Topical steroids (clobetasol propionate), oral corticosteroids (prednisone), oral antihistamines, and emollient agents can be used for treatment. Acneiform rash characterized by an eruption of papules and pustules, typically occurring in face, scalp, upper chest, and back is typically seen during MEK inhibitors therapy occurring in about 14% of patients [18, 22].

Papulopustular rashes can be treated with topical (clindamycin and erythromycin) and oral antibiotics (doxycycline and monocycline), topical (prednicarbate) and oral steroids (prednisone), oral antihistamines, and oral isotretinoin [18, 22].

Occasionally, keratosis pilaris-like eruptions may appear, and they can be managed with mild keratolytics such as urea cream or salicylic acid and topical steroids [19].

It has also been reported a nonspecific rash similar both clinically and histologically to idiopathic Grover's disease. This benign acantholytic dermatosis presents as scattered erythematous papules with or without crusting usually asymptomatic or only slightly pruritic. Lesions involve the trunk, the upper arms, and legs. It can be treated with moisturisers, topical steroids, and oral antihistamines or oral prednisone and acitretin in severe cases [19, 23].

Serious cutaneous rashes such as Stevens–Johnson syndrome and toxic epidermal necrolysis have been rarely reported [21].

### 2.3. Photosensitivity

Photosensitivity reaction represents one of the main adverse effects related to BRAF inhibitors, and it is experienced more frequently with the use of vemurafenib (22–67%) compared to dabrafenib (3–33%) [21]. Photosensitivity developed within days of drug initiation. Patients reported cutaneous eruptions on sun-exposed skin areas within hours of sun exposure. It is estimated that the cause of the reaction is related to the drug's chemical structure and UVA. Broad-spectrum sunscreens including UVA protection and protective clothing should be mandatory for patients [19, 21].

### 2.4. Palmarplantar Hyperkeratosis

Localized hyperkeratosis appeared as painful hyperkeratotic areas on points of pressure or friction mostly on the soles and less frequently on the palms [17]. It is observed in up to 60% in patients treated with vemurafenib and in up to 39% in patients treated with dabrafenib [21]. Differently from hand-foot skin reaction seen during chemotherapy, patients on BRAF inhibitors therapy report lesions with less inflammation, dysesthesia, blistering, desquamation, erythema, and ulceration [22, 24]. Plantar hyperkeratosis can be managed with regular use of urea cream and avoiding friction [19].

### 2.5. Verrucal Keratosis

The term verrucal keratosis is used to describe keratotic lesions as warts, keratoacanthomas, or nonspecific hyperkeratotic papules induced by BRAF inhibitors. These keratotic proliferative lesions develop as single lesions or a diffuse eruption at various anatomical sites, on both sun-damaged and non-sun-damaged skin with a median time to presentation of 11 weeks. Although verrucal keratoses are benign lesions, they should be monitored for changes such as rapid growth, pain, and erythema which are indicative signs of evolution into cutaneous SCC. Cryotherapy can be useful for small lesions. The use of acitrein as a chemopreventive drug has been beneficial [19, 23].

### 2.6. Squamous Cell Carcinoma

One of the most relevant adverse events reported in patients receiving BRAF inhibitors is the development of cutaneous tumors in the form of well-differentiated and keratoacanthoma-type squamous cell carcinomas. Clinically, these lesions appear as hyperkeratotic crateriform papules in various anatomical sites. SCCs have been reported to occur early during BRAF inhibitors therapy with a median time to onset of approximately 8 weeks. The reported incidence is 4–31% in patients treated with vemurafenib and 6–11% in patients treated with dabrafenib [19]. The treatment is simple surgical resection, and no dose adjustment of the therapy is required [19].

### 2.7. Other Dermatological Side Effects

Several changes affecting the hair follicle have been observed in patients during BRAF inhibitors treatment including alopecia, slower and thinner scalp hair growth, and structural changes in shape (from straight to curly) and in color (turn gray) of hair. These hair abnormalities are temporary, and they could spontaneously regress without therapy modifications. Although alopecia is a reversible effect, it has a strong impact on patient quality of life and may also lead to voluntary treatment disruption. Minoxidil solution until 3–6 months after therapy termination can be considered as a treatment for alopecia [19, 22].

Panniculitis on the lower extremities has been described with both vemurafenib and dabrafenib. It occurs as a painful, erythematous to livid, subcutaneous nodules, often accompanied by fever, chills, and arthralgia. Panniculitis responds to regular nonsteroidal anti-inflammatory drugs, and oral steroids (prednisolone) can also be considered for treatment [19, 22].

Although melanocytic proliferations are less frequent than squamoproliferative lesions, changes in preexisting nevi, new melanocytic nevi, and new primary melanomas have been reported in patients receiving BRAF inhibitors. The melanocytic proliferation under BRAF inhibitors treatment seems to be due to paradoxical activation of wild-type BRAF cells. However, it cannot be excluded that the onset of a new primary melanoma is related to the increased risk of developing a second primary melanoma among patients with a personal history of melanoma, rather than therapy. Therefore, a dermoscopic careful monitoring should be included in the follow-up of these patients [22].

Rare cases of basal cell carcinoma have been also described in patients under BRAF inhibitors treatment [21].

## 3. Targeting Immune Checkpoints

**Figure 2:**
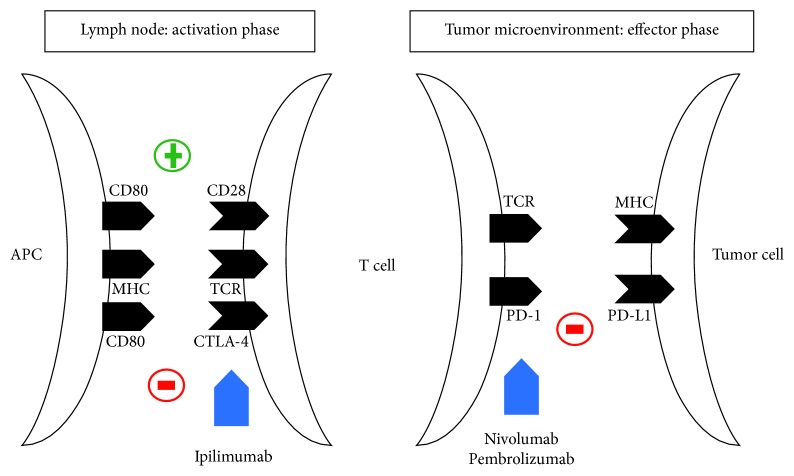
Immune-checkpoint inhibition of CTLA-4 and PD-1 pathways by antitumor immunotherapy.

Immunotherapy through immune-checkpoint inhibitors is an established treatment for advanced melanoma. The main immune-checkpoint-targeted molecules are CTLA-4 and PD-1, which are expressed on activated T-cells and are involved in the inhibition of the immune system. CTLA-4 is involved in the interaction between T-cells and antigen presenting cells (activation phase in lymphatic organs), whereas PD-1 mediates the interaction between T-cells and tumor cells (effector phase in peripheral tissues) [25] (Figure 2).

Currently, there are three monoclonal antibodies approved for the treatment of patients affected by metastatic melanoma: ipilimumab, which is an anti-CTLA-4 antibody, approved by FDA and EMA in 2011, and pembrolizumab and nivolumab, which are both anti-PD-1 antibodies, approved by FDA in 2014 and EMA in 2015. In 2016, FDA and EMA also approved the combination therapy of ipilimumab and nivolumab for the treatment of unresectable or metastatic melanoma [26].

Ipilimumab blocks the interaction of CTLA-4 with its ligands, CD80/CD86, allowing T-cell activation through reestablishment of CD28 to CD80/CD86 between the T-cell and antigen presenting cell. The inhibition of CTLA-4 signalling, through the activation of cytotoxic T-lymphocytes against cancer cells and the general increase in T-cell responsiveness, enhances the patient's antitumor immune response [27].

Pembrolizumab and nivolumab inhibit the interaction between PD-1 and PD-L1 enhancing antitumor responses, delaying tumor growth and facilitating tumor rejection [28, 29].

### 3.1. Cutaneous Side Effects of CTLA-4 and PD-1 Inhibitors

The alteration of the immune system induced by CTLA-4 and PD-1 inhibitors results in the development of various autoimmune manifestations referred as immune-related adverse events (irAEs). Dermatologic toxicity is very common, and it is potentially mediated by a shared antigen coexpressed by the dermoepidermal junction and tumor cells. Rash and pruritus are the most reported cutaneous side effects both in patients under treatment with ipilimumab and in patients treated with PD-1 inhibitors [30–34].

Cutaneous adverse events occurring during anti-PD-1 therapy are usually less severe and develop later than those seen with anti-CTLA-4 therapy (4–10 months compared with 3–6 weeks) [35].

Other common dermatologic adverse effects are lichenoid eruption, eczema, and vitiligo which are all mediated by lymphocyte damage confirming the immune-related mechanism.

Cutaneous side effects are mostly low grade and usually can be managed with symptomatic treatment that includes medium-to-high potency topical corticosteroids and oral antihistamines, while therapy interruption is rarely necessary [36].

Interestingly, skin immune-related events may be useful as visible clinical parameters of antimelanoma immunity and clinical response to checkpoint inhibitors. The development of vitiligo seems to be an indicator of antimelanoma immunity and improved survival [37].

### 3.2. Rash

Rash has been frequently reported in patients under treatment with immune-checkpoint inhibitors with an incidence of 15–40% [25]. The most common cutaneous toxicity of ipilimumab is a morbilliform rash which is characterized by erythematous macules and papules that typically involves the trunk and extremities sparing head, palms, and soles and is often associated with generalized pruritus [35]. It has a wide variable time to onset ranging from 3 weeks to 2 years after therapy initiation [38].

Other common subtypes of rash include lichenoid eruption and prurigo nodularis.

Lichenoid eruption has also been described in association with PD-1 inhibitor therapy. It predominantly appears on the chest and back as multiple erythematous and sometimes violaceous papules and plaques. Sometimes, lichenoid oral lesions may appear [35].

### 3.3. Eczema

Eczematous eruption is an adverse effect typically seen with anti-PD-1 therapy (up to 17% of patients) [39]. It occurs most commonly on the back and lower or upper limbs, less frequently on the face, chest, and abdomen. The lesions varied from classic eczema (ill-defined erythematous scaly lesions) to multiple nummular papules and are associated to pruritus in most cases [39].

### 3.4. Vitiligo

It is well known that the development of vitiligo in patients affected by advanced melanoma (stages III and IV) is associated with tumor regression and prolonged survival [40, 41]. Furthermore, it has been described that patients affected by vitiligo have a decreased risk of developing melanoma during life [42]. Vitiligo-like hypopigmentation has been frequently reported in patients treated with immune-checkpoint inhibitors with an incidence of 2–11% [25]. Vitiligo-like hypopigmentation lesions may appear on various areas of the body with a typical clinical presentation [39]. A recent meta-analysis suggests that vitiligo is associated with a better prognosis and it may be a marker of clinical response to immunotherapy [37].

### 3.5. Psoriasis

Some cases of psoriasis exacerbation have been reported in patients under treatment with nivolumab [43, 44]. T-cells, especially Th1 and Th17, play pivotal roles in pathogenesis of psoriasis. Anti-PD-1 agents upregulate Th1 and Th17 cells which induce the release of IL-17 and IL-22 leading to inflammation and keratinocyte proliferation. This mechanism supports the potentially exacerbating role of PD-1 inhibitors in psoriasis.

### 3.6. Autoimmune Blistering Diseases

Several cases of bullous pemphigoid and bullous pemphigoid-like skin lesions have been described in patients under treatment with anti-PD-1 agents [45–48], whereas rare cases of bullous skin lesions have been reported after anti-CTLA-4 antibodies [49, 50]. Systemic corticosteroids are the first-line treatment.

Bullous pemphigoid has also been reported as a paraneoplastic manifestation of melanoma. The exact mechanism of the paraneoplastic phenomenon remains poorly understood, but it has been described that BP180 is expressed in melanoma cells and not in normal melanocytes [51]. While most cases of bullous pemphigoid have been reported in patients treated with anti-PD-1 agents, it is possible that the use of immune-checkpoint inhibitors induces a loss of self-tolerance responsible of the appearance of bullous skin lesions.

### 3.7. Other Dermatological Side Effects

Less common cutaneous adverse events afflicting patients during immunotherapy include xerosis, photosensitivity reactions, pyoderma gangrenosum-like ulcerations, Sweet syndrome, cutaneous sarcoidosis, alopecia, actinic keratosis, squamous cell carcinomas, seborrheic keratosis, toxic epidermal necrolysis, and severe drug rash with systemic symptoms and eosinophilia [26, 35].

## 4. Conclusions

Targeted therapies have a crucial role in patients with advanced melanoma. They have significant benefits in the prognosis, but they are frequently associated with cutaneous side effects that may affect quality of life of patients. Cutaneous adverse events are usually low grade and manageable. Understanding and managing skin toxicity could improve the quality of life and prevent the interruption of the tumor therapy leading to a better clinical outcome. Although current targeted therapies have been shown to reduce melanoma mortality, advanced melanoma is still a significant therapeutic challenge to clinicians. New therapeutic approaches are currently being developed, and they will probably include combination therapies as the association of BRAF inhibitors and immune-checkpoint inhibitors. New potential side effects will likely emerge introducing these novel combination therapies.

## Figures and Tables

**Table 1:** Cutaneous side effects observed during targeted therapy (BRAF and MEK inhibitors) and immunotherapy (CTLA-4 and PD-1 inhibitors) and their management.

Target	Skin toxicity	Management
	Skin rash (maculopapular)	Topical steroids (clobetasol propionate); oral corticosteroids (prednisone); oral antihistamines; emollient agents
BRAF inhibitors (i) Vemurafenib (ii) Dabrafenib
	Photosensitivity	Avoid sun (broad-spectrum sunscreens that cover UVA spectrum, protective clothing)
	Palmarplantar hyperkeratosis	Urea cream; avoid friction
	Verrucal keratosis	Cryotherapy; monitor for changes suggestive of SCC; acitrein as a chemopreventive drug
	Squamous cell carcinoma, alopecia, and hair modifications	Excision, minoxidil 2%
	Panniculitis	Nonsteroidal anti-inflammatory drugs; oral steroids (prednisolone)
	Melanocytic proliferation	Dermoscopic monitoring; radical surgery for melanomas; education on photoprotection and self-skin examination
	BCC	Excision

MEK inhibitors	Acneiform rash (papulo-pustular)	Topical antibiotics (clindamycin, erythromycin); oral antibiotics (doxycycline, monocycline); topical steroids (prednicarbate); oral steroids (prednisone); oral antihistamines; oral isotretinoin
(i) Trametinib		
(ii) Cobimetinib		

CTLA-4 inhibitors	Rash (maculopapular, lichenoid eruption), eczema	Medium-to-high potency topical (and sometimes oral) corticosteroids; antihistamines
(i) Ipilimumab		

PD-1 inhibitors	Vitiligo, psoriasis, autoimmune blistering disorders	
(i) Nivolumab		
(ii) Pembrolizumab		
